# Synthesis of Epoxidized Soybean Oil as a Bio-Based Chain Extender for Recycled Polyethylene Terephthalate and Optimization of the Extrusion Process for Its Performance

**DOI:** 10.1021/acsomega.5c08261

**Published:** 2025-12-26

**Authors:** Mahdieh Nasehifar, Abbas Rezaee Shirin-Abadi, Michael Enayati

**Affiliations:** † Department of Polymer & Materials Chemistry, Faculty of Chemistry & Petroleum Sciences, 48512 Shahid Beheshti University, Tehran 1983969411, Iran; ‡ Center for Materials and Manufacturing Sciences, Department of Chemistry and Physics, 8025 Troy University, Troy, Alabama 36082, United States

## Abstract

Polyethylene terephthalate (PET), widely used in textiles and packaging, loses its mechanical strength during melt processing and recycling due to chain scission, resulting in reduced intrinsic viscosity (IV). Epoxidized soybean oil (ESBO), a biobased and ecofriendly modifier, offers a sustainable alternative to petroleum-derived chain extenders (CEs) to recover the IV. This study explores the effect of ESBO on the IV and mechanical properties of recycled PET (r-PET) via reactive extrusion using a single screw extruder. Various ESBO concentrations were tested, and it was found that 2.0 wt % ESBO with a 10 min residence time (B_2–10min_) achieved the best enhancement in IV and mechanical performance of the r-PET. The findings suggest that optimizing parameters such as CE loading and residence time can further improve ESBO’s efficiency in restoring PET properties. Characterization by viscometry, Fourier transform infrared spectroscopy, nuclear magnetic resonance spectroscopy, thermogravimetric analysis, field emission scanning electron microscopy, X-ray diffraction, and tensile strength confirmed that ESBO effectively increased the molecular weight, thermal stability, and mechanical properties of r-PET under optimal conditions.

## Graphical abstract

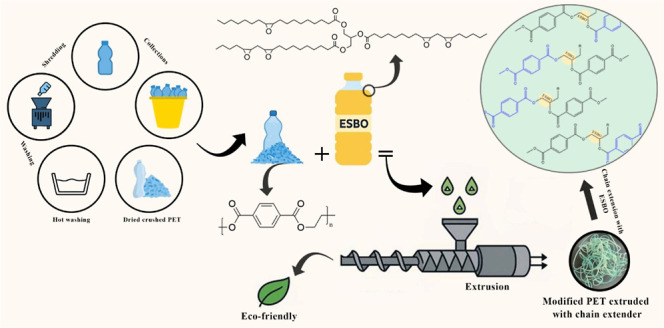

## 1 Introduction

Polyethylene terephthalate (PET) is one of the most used commodity plastics in which its popularity stems from its impressive chemical and mechanical properties, such as durability, lightweight, transparency, and excellent barrier performance.^1,2^ These features make PET ideal for various applications, from food and beverage packaging to textiles and automotive parts.^1,2^ With increasing consumption, there has been growing interest in recycling of PET as it represents 8% of global solid waste by weight and 12% by volume.^3–5^ By developing effective recycling processes, the adverse environmental impacts of PET can be minimized and its sustainability can be enhanced.^6^ Among the key recycling methods for polymers, mechanical and chemical recycling are significant.^7–9^ Mechanical recycling reprocesses PET into new products using established industrial equipment such as extruders, while chemical recycling depolymerizes PET into monomers or valuable chemicals, enabling the production of high-purity raw materials. A clear understanding of the advantages and limitations of these established pathways is essential for improving PET recycling.^10^ Despite the widespread adoption of mechanical recycling, challenges such as feedstock contamination, degradation of polymer, loss of mechanical properties, and a limited number of recycling cycles emphasize the need for optimized recycling strategies and further research on PET recovery. The mechanical recycling of PET by melt reprocessing is currently the method of choice by industry due to its relative simplicity, low capital investment, utilization of established equipment, and flexibility regarding feedstock volume.^11^ Additionally, this method presents minimal adverse environmental impacts, making it a prudent choice for the recycling of PET materials.^12^ Mechanical recycling consists of multiple stages, including the collection of waste, sorting, washing, grinding, and finally, converting the processed materials into recycled pellets.^13^ During the high-temperature melt reprocessing of PET at around 270 °C in an extruder, the polymer experiences various forms of degradation—chemical, mechanical, thermal, and oxidative. This degradation results in a decrease in the molecular weight, which subsequently lowers its intrinsic viscosity (IV), melt strength, and mechanical properties. As a consequence, the recycled PET (rPET) is less suitable for many applications.^14,15^

To counteract PET degradation during its processing, many studies suggest that incorporating chain extenders (CEs) can effectively increase molecular weight and restore essential properties.^16,17^ CEs are multifunctional and have the ability to react with the carboxyl and hydroxyl end groups present in PET that lead to an increase in the molecular weight (and IV) of the polymer chains, resulting in improved mechanical properties such as tensile strength, flexibility, and impact resistance.^18^ The CE reaction with PET end groups is fast, effective, and straightforward and provides a cost-effective approach to the PET chain scission problem during processing. The chain extension process can be easily performed using standard processing equipment, including internal mixers, continuous mixers, extruders, and even injection molding machines.^19^ Epoxides, renowned for their high reactivity, are employed as the main functional groups in CEs for PET recycling processes. A notable advantage of epoxides is their compatibility with food grade materials, making them a good choice in the packaging industry.^20^ Epoxides are reactive three-membered cyclic ethers, which have the ability to react with proper functional groups through ring-opening reactions. As shown in Scheme 1, the ring-opening reaction of epoxides with PET can be classified into two distinct pathways. The first pathway involves a reaction between the epoxide ring and the carboxyl groups present in PET, while the second pathway proceeds through a reaction with hydroxyl groups.^21,22^

**Scheme 1:**
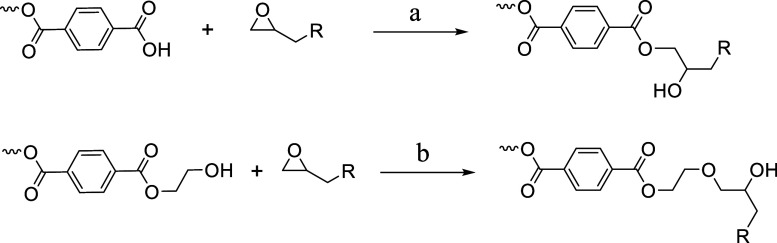
Chain Extension Mechanism Involving the Reaction of the Epoxide Functional Group with the PET End Group: (a) Reaction between the Carboxyl End Group of PET and Epoxide; (b) Reaction between the Hydroxyl End Group of PET and Epoxide

Industrial CEs are usually copolymers of styrene and acrylic monomers with desirable functionality that mostly contain epoxy groups.^23^ These synthetic CEs, such as Joncryl, generally show higher reactivity due to multiple functional groups. However, biobased alternatives like plant-derived oils offer environmentally friendly alternatives by reducing dependency on petroleum-based chemicals and minimizing toxic byproducts. Although their reactivity might be lower, their performance remains acceptable, making them promising candidates for sustainable PET modification. Considering the importance of using more sustainable products in polymer recycling industries, the utilization of natural materials as CEs by epoxide functionalization stands out as a reasonable approach for more sustainable PET mechanical recycling. Soybean oil, which is considered one of the most widely used and cost-effective vegetable oils, can be obtained through mechanical methods or extraction using organic solvents. Epoxidized soybean oil (ESBO) serves as an effective additive with numerous benefits, particularly in enhancing the properties of polymers and improving recycling processes.^24^ ESBO’s ability to increase mechanical and thermal stability makes it valuable in creating high-performance materials.^25,26^ Extrusion serves as an effective method for recycling polymers; however, there are challenges in maintaining material properties throughout the process. By carefully controlling the extrusion parameters during the chain extension, it is possible to maximize the improvement in the properties of rPET. Continuous evaluation and optimization of extrusion parameters are crucial for improving the quality and usability of recycled products, especially in the case of rPET.^27^

In this study, ESBO was synthesized as a CE for rPET with rather low IVs and its effect has been studied during extrusion. In the initial phase, extruder parameters such as temperature, screw speed, and continuous extrusion (constant processing time of one min) or batch extrusions (timed extrusions with different processing time) were examined to optimize the interaction between the CE and rPET, in order to maximize the effect of CE. Furthermore, the influence of residence time and ESBO loading during reactive extrusion was systematically investigated. Through this approach, the mechanical recycling process can be directed toward minimizing the negative effects on rPET while enhancing its overall properties. Our main focus was process optimization, lowering energy consumption, and increasing the efficiency of PET mechanical recycling. Achieving these goals is expected to yield significant economic benefits while simultaneously contributing to environmental sustainability due to using a biobased CE.

## 2 Experimental Section

### 2.1 Materials

Two samples of sorted, cleaned, shredded, hot washed polyethylene terephthalate flakes (r-PET) with an IV range from 0.330 to 0.450 dL/g (rPET sample 1) and 0.390 to 0.490 dL/g (rPET sample 2) were supplied from Pishtazan Pardis Persin (P3 Chemical Co., Iran). These samples contain around 10% pale blue flakes mixed with transparent rPET flakes. A 60:40 mixture of phenol and 1,1,2,2-tetrachloroethane (both from Merck) was used to prepare a 0.50 wt % solution of PET samples for viscometry measurements. Freshly prepared soybean oil was purchased from local store and used to synthesize ESBO without further purification. Formic acid (85 wt %) and hydrogen peroxide’s aqueous solution (60 wt %) were purchased from Merck and were used for the epoxidation of soybean oil, whereas sodium hydroxide and hydrochloric acid (Merck) were used in the hot wash process. The materials used for assessing the epoxy concentration including glacial acetic acid, tetraethylammonium bromide (anhydrous crystals), perchloric acid (0.10 N in glacial acetic acid), acetic anhydride, methylene chloride, crystal violet indicator, and potassium hydrogen phthalate were all prepared from Merck. Deionized water was used for the acid number measurements.

### 2.2 Synthesis of Epoxidized Soybean Oil (ESBO)

Epoxidized soybean oil was synthesized by reacting a mixture of 10.0 g of soybean oil with 0.60 g of formic acid at 60 °C in a 150 mL round-bottom flask, followed by the gradual addition of 4.0 g of 60 wt % hydrogen peroxide solution and magnetic stirring. Throughout the reaction, careful control of the hydrogen peroxide addition rate is needed, while the temperature should be carefully maintained at 60 °C. Failure to control this rate may lead to an increase in reaction temperature, potentially resulting in the degradation of the epoxide groups and/or side reactions. The optimal reaction time is estimated to be around 6 h.^28^ The chemical structure of synthesized epoxidized soybean oil is shown in Scheme 2.

**Scheme 2:**
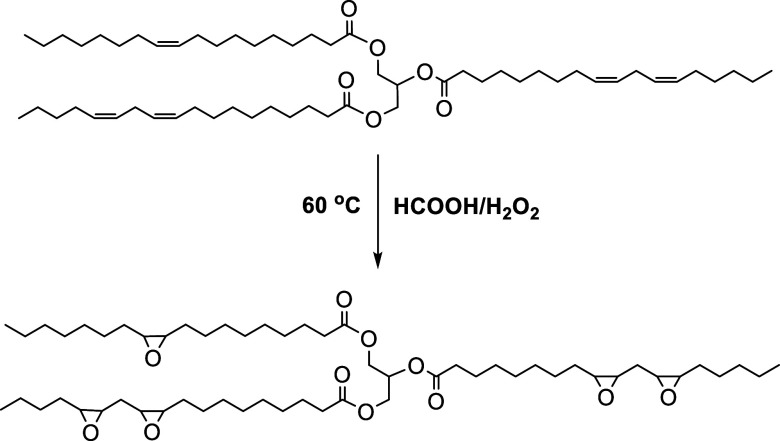
Synthesis of ESBO from Soybean

### 2.3 Sample Preparation for the Extruder

Prior to mixing with the CE, the rPET samples were kept in an oven at 150 °C for a duration of 4 h. Subsequently, the samples are cooled to room temperature, and then different concentrations of acetone-diluted ESBO were added. Before extrusion, the samples were placed in an oven at 50 °C for 10 min to ensure the removal of acetone and then processed in the extruder.

### 2.4 Extruder Specification

The extruder used in this study was a single-screw extruder in which the rPET reaction with the CE was conducted in both continuous and batch (timed) modes. For the batch samples, to achieve a longer residence time within the extruder, the screw rotation was halted at a specified temperature, a method intended to enhance the ESBO reaction. This approach aimed to mitigate, to some extent, the issue of the potential incomplete reaction of ESBO with rPET which is attributed to the lower mixing efficiency and the short residence time of the single-screw extruder. The conditions of the extruder for the reactive extrusion of ESBO with rPET are summarized in Table 1.

**Table 1:** Extruder Conditions for Reactive Extrusion of ESBO with rPET

parameter	value
zone 1: extruder temperature (below the hopper) [°C]	180
zone 2: barrel temperature [°C]	260
zone 3: barrel temperature [°C]	280
zone 4: die temperature [°C]	240
screw speed [rpm]	27

### 2.5 Acid Number Measurement

To ascertain the acid number (carboxyl end groups) of PET samples, the polymer sample is solubilized rapidly in benzyl alcohol at an elevated temperature (about 203 °C) for around 105–110 s.^29^ The resultant solution was then quickly mixed with room-temperature chloroform to form a metastable solution or dispersion, which makes the acid groups ready to be titrated with a base. Subsequently, this mixture was titrated with 0.10 N NaOH in benzyl alcohol, using phenol red as the indicator until a stable pink end point is sustained for 10 s. The titrant volume is adjusted for blank contributions (benzyl alcohol–chloroform system) and degradation, with the final value represented as equivalents of carboxyl groups (moles) per 10^6^ g of the polymer, calculated from eq 1.

1 $$AN=\frac{\left(V-V_{b}\right)\times N\times 10^{6}}{W}$$

where *V* = volume of NaOH solution used for the sample (mL), *V*_b_ = volume of NaOH solution used for the blank (mL), *N* = normality of NaOH solution (N), and *W* = weight of polymer sample (*g*). This methodology produces dependable outcomes owing to meticulous temperature regulation, exact dissolving time, swift end point identification, and degradation adjustments.

### 2.6 Characterization

The epoxy number of the epoxidized soybean oil was assessed in accordance with ASTM D1652-11, while the rPET viscometry measurements were evaluated following ASTM D4603-18 using an Ubbelohde 1B viscometer operated in a bath at 30.0 °C, with a mixture of phenol and tetrachloroethane (60:40 by weight) as the solvent. A solution with a concentration of 0.50 wt % by weight of PET was required, with dissolution temperatures maintained between 90 and 110 °C for 15–30 min, depending on each specific sample. First, the inherent viscosity was determined, and then the IV ([η]) was calculated using the Billmeyer equation. Three viscosity measurements were taken for each sample, and the average is reported. A Thermo Nicolet NEXUS 470 instrument (Waltham, Massachusetts, USA) with an attenuated total reflectance accessory was used to obtain the Fourier transform infrared (FTIR) spectra. A Bruker Avance DPX nuclear magnetic resonance (NMR) spectrometer (Billerica, Massachusetts, USA) was employed to acquire ^1^H NMR spectra at 300 MHz in a CDCl_3_ solution with tetramethylsilane (TMS) as an internal standard at 25 °C to verify the chemical structure of the epoxidized soybean oil. Thermal stability was tested using thermogravimetric analysis (TGA) with Dama Pajouh Arvin (Tehran, Iran) equipment. Around 5 mg of sample was heated at a rate of 10 °C/min from 25 to 600 °C in a nitrogen atmosphere. The crystal structures of PET were analyzed using a powder X-ray diffraction (XRD) diffractometer (STOE IPDS II, Darmstadt, Germany) with Cu Kα radiation (λ = 1.540598 Å) at 40 Kv. A scan speed of 0.06 s/step was used to get the diffraction patterns, which spanned a 2θ range of 10–80 degrees. Field emission scanning electron microscopy (FE-SEM) analysis of the rPET samples were performed using a Tescan Mira II microscope. Images were captured at 100 and 20 μm magnifications with an accelerating voltage of 15 kV. The tensile specimens were prepared in accordance with ASTM D882-12 and subjected to a tensile test at 25 °C using the Santam STM-20 apparatus. The measurements were conducted under controlled conditions to ensure the repeatability and reliability of the results. Differential scanning calorimetry (DSC) was performed utilizing a Dama Pajouh Arvin apparatus (Iran) throughout the temperature range 25-300 °C, at a heating rate of 10 °C/min. The degree of crystallinity (χ_c_) of the samples was evaluated using the melting enthalpy (Δ*H*_m_), cold crystallization enthalpy (Δ*H*_c_), and predicted melting enthalpy of 100% crystalline PET (Δ*H*_m_^0^), which is considered to be 140.1 J/g.^30^ Eq 2 was used to calculate the crystallinity

2 $$\chi_{c}=\frac{\Delta H_{m}-\Delta H_{c}}{\Delta H_{m}^{0}}\times 100$$

## 3 Results and Discussion

### 3.1 FTIR Spectroscopy Analysis

Figure 1 shows the ATR-FTIR spectra of soybean oil and ESBO, revealing the structural transformations induced by the epoxidation process. In the spectrum of soybean oil, a strong absorption band at approximately 1740 cm^–1^ corresponds to the stretching vibration of the ester carbonyl (C=O) functional group, which is a characteristic feature of triglycerides. The peak observed around 1650 cm^–1^ is attributed to the stretching vibration of C=C bonds, indicative of the unsaturation (double bonds) within the fatty acid chains.^31^ Additionally, the absorption bands in the 2800–3000 cm^–1^ region arise from the stretching vibrations of aliphatic C–H bonds, while the peak at 3010 cm^–1^ is due to the vinylic hydrogens. Following epoxidation, notable spectral alterations are observed. The reduction of the peak at 1650 cm^–1^ indicates the conversion of carbon–carbon double bonds into epoxy rings.^32^ Also, the peak at around 3010 cm^–1^ which related to the olefinic hydrogens reduced dramatically in the ESBO.^33^ Furthermore, the emergence of new absorption bands in the 820 cm^–1^ is indicative of the characteristic vibrations of the oxirane (C–O–C) functional group.^34^ Notably, the persistence of the ester carbonyl peak at 1740 cm^–1^ confirms that the triglyceride backbone remains intact. These spectral modifications unequivocally validate the successful epoxidation of soybean oil, resulting in ESBO with enhanced reactivity and functional versatility.^35^

**Figure 1:**
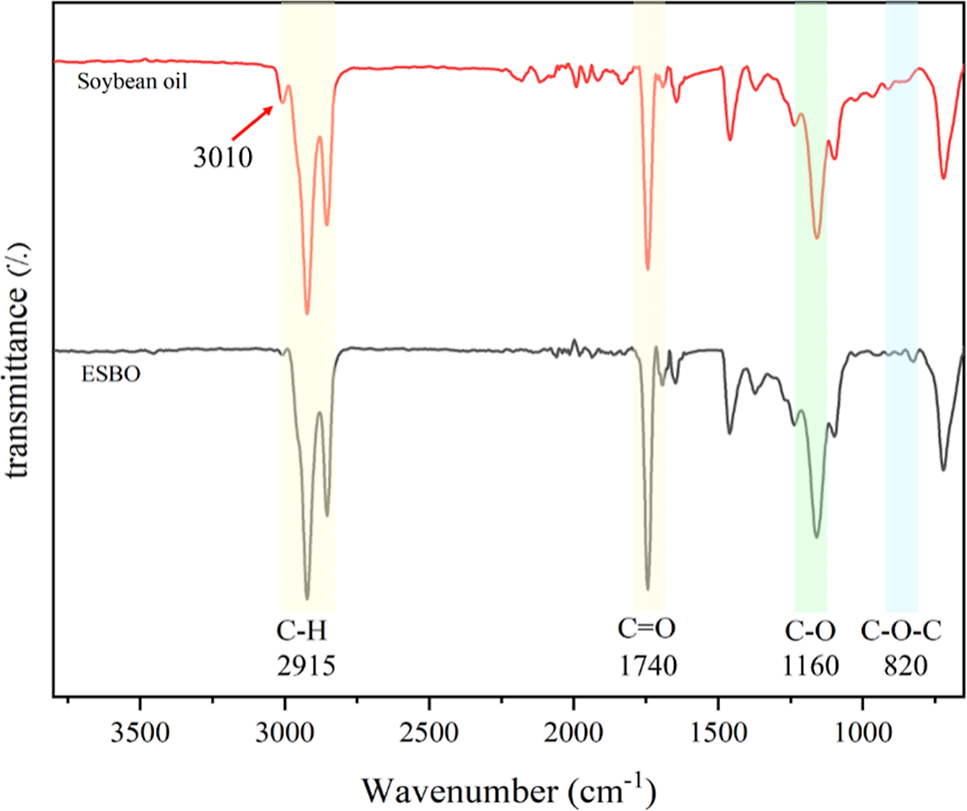
ATR-FTIR spectra of (a) soybean oil (b) ESBO with labeled peaks: vinylic hydrogens ∼3010 cm^–1^, aliphatic C–H ∼2915 cm^–1^, C=O ∼1740 cm^–1^, C–O ∼1160 cm^–1^, and C–O–C ∼820 cm^–1^. Spectra are normalized at the carbonyl peak (∼1740 cm^–1^).

### 3.2 NMR Spectroscopy Analysis

The structural transformation of soybean oil during epoxidation was also investigated by using ^1^H NMR spectroscopy, as illustrated in Figure 2. A distinct signal was observed in the spectrum of the unmodified soybean oil at approximately 5.2–5.5 ppm, which corresponds to the vinylic protons of the internal double bonds. The utilization of carbon–carbon double bonds during the epoxidation reaction was indicated by the significant decrease in the intensity of this peak. At the same time, new resonances were observed in the 3.0–3.2 ppm range, which are indicative of protons that are associated with the epoxide rings.^36,37^ The successful introduction of epoxy functionalities is confirmed by these spectral changes. Furthermore, the signals associated with the glycerol backbone, specifically the methylene protons, which were observed at 4.1–4.3 ppm, respectively, remained unaltered, indicating that the primary ester structure was preserved during the epoxidation process.^38,39^

**Figure 2:**
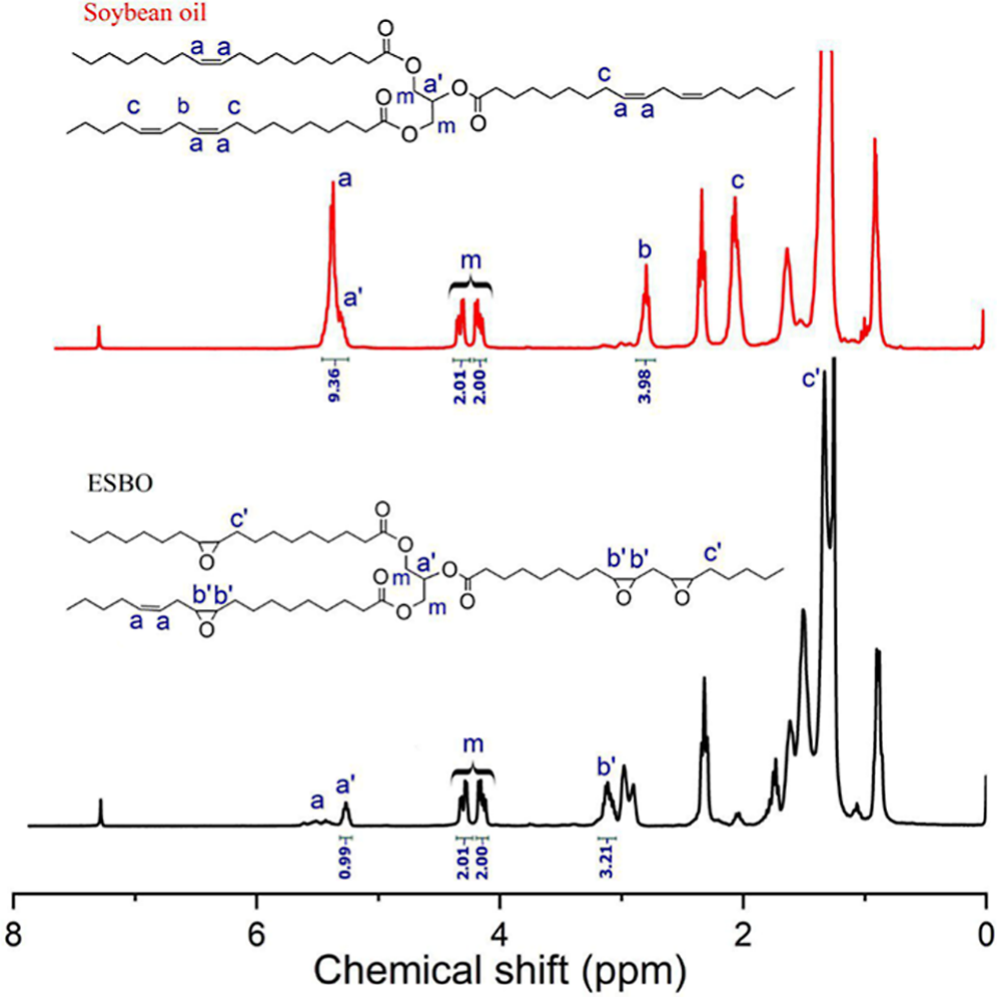
^1^H NMR spectra of soybean oil and ESBO in CDCl_3_.

### 3.3 Analysis of Epoxy Number in ESBO

The epoxy number of epoxidized soybean oil was determined by following the ASTM D1652-11 method. For the titration process, a solution was prepared by dissolving 100.0 g of tetraethylammonium bromide in 400 mL of acetic acid. A 0.10 wt % solution of crystal violet was also made by dissolving 0.10 g of crystal violet in 100 mL of acetic acid. Approximately 0.3 to 0.4 g of epoxidized soybean oil was weighed and dissolved in 10 mL of dichloromethane. To this solution was added 10.0 mL of the tetraethylammonium bromide solution, followed by the addition of 8 to 10 drops of the crystal violet solution to serve as an indicator. The epoxy number was then determined by titrating the mixture with 0.10 N perchloric acid in glacial acetic acid. Equation 3 provides the formula for determining the epoxy number (*O*) of epoxidized soybean oil.

3 $$O=\frac{1.6\;N\times V}{W}$$

where *N* is the normality of the titrant (0.10), *V* is the volume consumed (mL), and *W* is the weight of the sample (g). The epoxy number obtained by using this equation was 6.43.

### 3.4 Viscosity Analysis of ESBO-Treated rPET Samples

In order to assess the efficiency of the ESBO in increasing the IV of the extruded rPET, we have run several continuous (C) and batch (B) extrusions of rPET (samples 1 and 2) with and without ESBO as a CE. The ratios of ESBO to rPET in these experiments were 0, 1.0, 2.0, 3.0, and 4.0 wt %, and the time for continuous samples (C) was around 1 min (due to the extruder length and speed) while the time for batch (timed) samples (B) was 10 and 15 min. The values obtained from viscosity measurements under different conditions are reported in Table 2. It should be noted that ESBO loading below 1 wt % has failed to show a successful reaction or consistent results due to the insufficient concentration of epoxy in the structure and/or poor distribution of the ESBO. It should be noted that for each batch sample, also a control (a blank sample without ESBO) was run, and the corresponding increase in the IV of the sample is shown in Figure 3. Since different rPET with different initial IV were used here, the change in IV for each sample, either continuous or batch, is compared with its own control (Table 2 and Figure 3).

**Table 2:** Processing Conditions and Viscometry Results for Continuous (C) and Batch (Timed) Samples (B) with and without ESBO^a^

sample	*W*_ESBO_	*W*_PET_	ESBO (wt %)	IV (dL/g)	sample	W_ESBO_	W_PET_	ESBO (wt %)	IV (dL/g)
(a) rPET sample 1									
C_0–1min_	0	150 g	0	0.330 ± 0.009	B_1–10min_	1.5 g	150 g	1.0	0.378 ± 0.014
C_1–1min_	1.5 g	150 g	1.0	0.352 ± 0.011	B_1–15min_	1.5 g	150 g	1.0	0.175 ± 0.008
C_0–1min_	0	150 g	0	0.310 ± 0.012	B_2–10min_	3.0 g	150 g	2.0	0.565 ± 0.011
C_2–1min_	3.0 g	150 g	2.0	0.360 ± 0.014	B_2–15min_	3.0 g	150 g	2.0	0.681 ± 0.013
C_0–1min_	0	150 g	0	0.448 ± 0.006	B_3–10min_	4.5 g	150 g	3.0	0.511 ± 0.022
C_3–1min_	4.5 g	150 g	3.0	0.499 ± 0.010	B_3–15min_	4.5 g	150 g	3.0	0.524 ± 0.018
C_0–1min_	0	150 g	0	0.402 ± 0.004	B_4–10min_	6.0 g	150 g	4.0	0.489 ± 0.008
C_4–1min_	6.0 g	150 g	4.0	0.444 ± 0.016	B_4–15min_	6.0 g	150 g	4.0	0.508 ± 0.014
(b) rPET sample 2									
C_0–1min_	0	150 g	0	0.413 ± 0.017	B_1–10min_	1.5 g	150 g	1.0	0.433 ± 0.014
C_1–1min_	1.5 g	150 g	1.0	0.410 ± 0.029	B_1–15min_	1.5 g	150 g	1.0	0.220 ± 0.009
C_0–1min_	0	150 g	0	0.420 ± 0.021	B_2–10min_	3.0 g	150 g	2.0	0.664 ± 0.007
C_2–1min_	3.0 g	150 g	2.0	0.512 ± 0.017	B_2–15min_	3.0 g	150 g	2.0	0.689 ± 0.019
C_0–1min_	0	150 g	0	0.391 ± 0.005	B_3–10min_	4.5 g	150 g	3.0	0.606 ± 0.012
C_3–1min_	4.5 g	150 g	3.0	0.515 ± 0.019	B_3–15min_	4.5 g	150 g	3.0	0.677 ± 0.015
C_0–1min_	0	150 g	0	0.450 ± 0.021	B_4–10min_	6.0 g	150 g	4.0	0.489 ± 0.011
C_4–1min_	6.0 g	150 g	4.0	0.555 ± 0.010	B_4–15min_	6.0 g	150 g	4.0	0.651 ± 0.009

a C_X‑Y_: continuous extrusion, samples exited the extruder continuously and without extra residence time; B_*X*–*Y*_: batch (timed) extrusion, samples have been extruded and kept in the extruder for the designated processing time. *X*: amount of CE (wt %) utilized; *Y*: extruder residence time (min).

**Figure 3:**
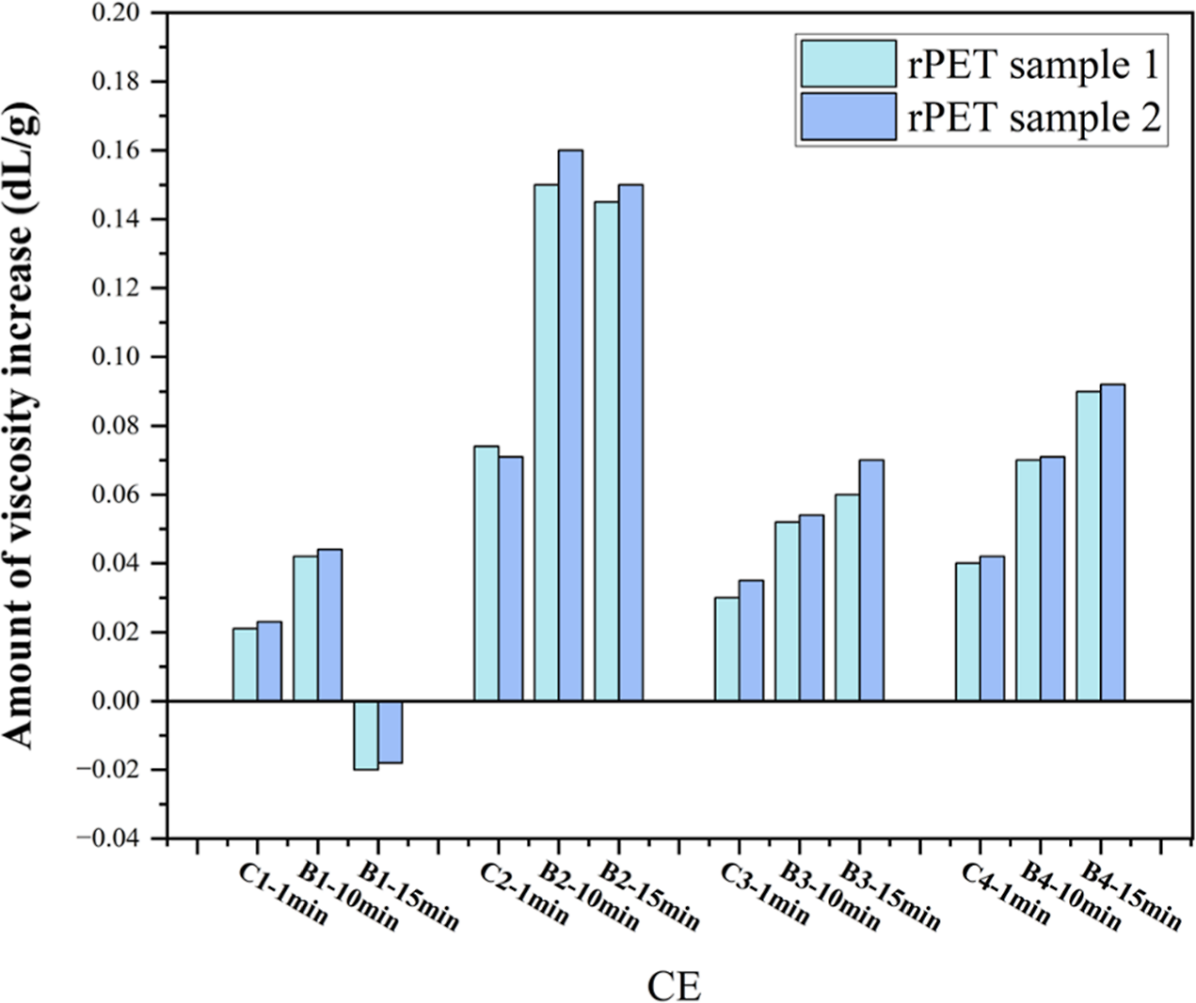
IV increase (or decrease) at different ratios of CE compared to the control (0% ESBO) for both rPET sample 1 and rPET sample 2.

Figure 3 shows the amount of IV increase for each sample due to the incorporation of the ESBO in addition to the effect of the extrusion time. As it can be seen in this Figure 3, the highest level of effectiveness, based on the measured IV, was achieved at a concentration of 2.0 wt % ESBO in 10 and 15 min, compared to the control. We hypothesize that at ESBO concentrations higher than 2.0 wt %, the extra ESBO act like a low-molecular-weight diluent and this effect canceled its chain extension effect. Another possible scenario is the possibility of gelation of PET at the higher ESBO ratio, and since the gel part will not dissolve in the solvent mix for viscometry measurement, a lower IV will be recorded.

We also analyzed the samples visually. As shown in Figure 4, the effect of the CE on the appearance and profile’s diameter of the PET samples can be observed. The blue color of the samples is due to the original r-PET sample, which experienced slight color changes as a result of the reaction with ESBO, which is pale yellow. The most noticeable effect is clearly seen in sample B_2–10min_. The increase in IV for sample B_2–10min_ was approximately 0.140 dL/g, which was determined through viscometry and comparison with the blank rPET sample. To analyze the influence of extrusion time on the efficacy of the CE, longer residence times of ESBO and rPET in the extruder were also examined. This assessment was conducted at processing times of 20, 25, 30, and 60 min to study the impact of longer residence time (Figure 5).

**Figure 4:**
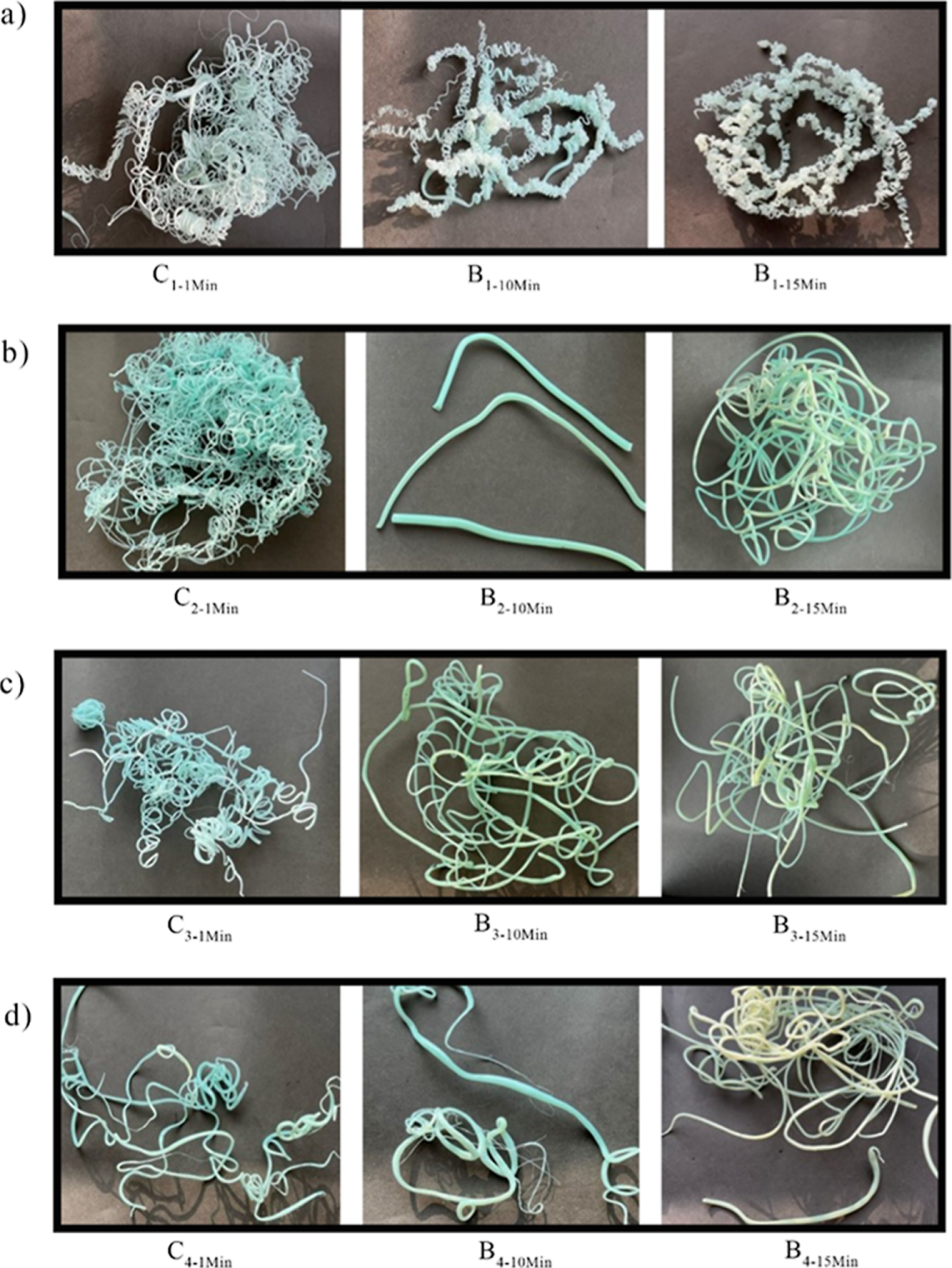
Shape and profile’s diameter of extruded samples with different CE contents and processing conditions: (a) 1.0 wt % CE, (b) 2.0 wt % CE, (c) 3.0 wt % CE, and (d) 4.0 wt % CE, at 1 min mixing (continuous), 10 min mixing, and 15 min mixing, respectively. Photos are 25 cm × 25 cm.

**Figure 5:**
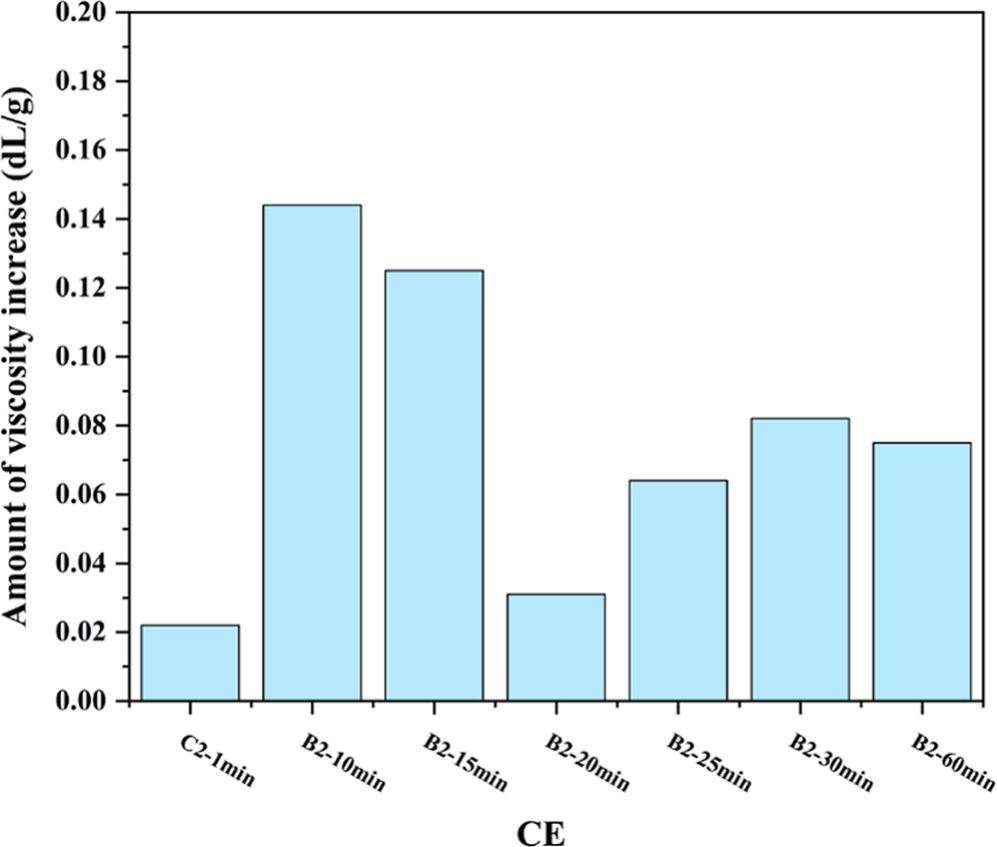
Effectiveness of the CE at 2.0 wt % at different times compared to the control sample (sample with 0% ESBO and the same extruder time).

Increasing the residence time in the extruder can have two opposing effects on IV. On one hand, a longer residence time, compared to continuous mode, enhances the reaction of the CE with the PET. On the other hand, extended residence times can lead to thermal degradation due to the high temperature within the extruder. Thermal degradation can cause chain scission and a subsequent IV loss and increase in acid end groups. The extent to which longer residence times affect IV of PET, with and without ESBO, is shown in Table 3.

**Table 3:** Viscometry Measurement of Some Samples with and without ESBO at Longer Residence Time^a^

samples	residence time (min)	ESBO (wt %)	IV (dL/g)	IV increase compared to control (dL/g)
B_0–20min_	20	0	0.447 ± 0.017	-
B_2–20min_	20	2.0	0.478 ± 0.027	0.031
B_0–25min_	25	0	0.367 ± 0.010	-
B_2–25min_	25	2.0	0.431 ± 0.014	0.064
B_0–30min_	30	0	0.420 ± 0.015	-
B_2–30min_	30	2.0	0.502 ± 0.018	0.082
B_0–60min_	60	0	0.207 ± 0.005	-
B_2–60min_	60	2.0	0.282 ± 0.011	0.075

a The blank rPET used for this experiment has an initial IV of 0.510 ± 0.022 dL/g.

Several parameters can affect the increase in IV, but two major parameters exist with opposing effects. As mentioned, increasing the residence time may enhance the reaction between the CE and PET; however, the prolonged residence time might also result in thermal deterioration of PET. Consequently, an optimal equilibrium between extending the residence time and preserving the structural integrity of the polymer chains must be attained to obtain optimal properties. The thermal degradation of PET chains can result in a reduced molecular weight and mechanical properties of the polymer while influencing the concentration of functional groups. During thermal deterioration, particularly at elevated temperatures, hydroxyl and carboxylic acid groups may be generated. The generation of these groups as the reactive agent during the PET processing may promote side reactions with the CE (Scheme 1). Thus, although heat deterioration may compromise structural integrity, the formation of hydroxyl and acidic groups can potentially enhance chemical processes, ultimately resulting in improved CE’s efficiency and the stabilization of the final product. Consequently, despite significant structural degradation, the efficacy will remain elevated after 1 h. It can be seen in Figure 5 that in the initial stages, the effect of the CE is relatively insignificant. With increasing reaction time, an improvement in properties is gradually observed; however, the greatest effect of this CE is observed in 10 min (Figure 5). In 20 min, more degradation occurs in the control sample, and the least effect is recorded. After this time, due to the creation of hydroxyl and acidic groups in the structure, the polymer has a better ability to react with ESBO and, as shown in Figure 5, the reaction will move toward a slight increase in the IV.

### 3.5 XRD Analysis of PET Samples with 2.0 wt % Chain Extender under Varying Conditions

XRD analysis was conducted to investigate the effect of the CE on the crystallinity, as shown in Figure 6. Under continuous conditions, an increase in crystallinity was observed compared to the control sample, attributed to the reaction with the CE, which created a limited order and results in increasing crystallinity.^40^ This limited molecular interaction facilitates the formation of localized crystalline regions, resulting in a slight increase in the crystallinity. However, at longer reaction times of 10 and 15 min, further interaction with the CE led to a decrease in order and a transition toward an amorphous structure. Figure 6 illustrates that extended residence time of PET in the extruder (25–30 min) leads to thermal degradation of its structure. After breaking down and getting more acidic groups, the material can react again with the CE, which makes the ESBO structure being a part of the crystalline phase. Consequently, greater order is observed in comparison to the 10- and 15 min samples, leading to a marginal increase in crystallinity, which can be attributed to the formation of limited masses around the CE. These findings suggest that ESBO plays a complex, time-dependent role in the crystallization behavior of recycled PET by balancing chain extension with the effects of thermal degradation.

**Figure 6:**
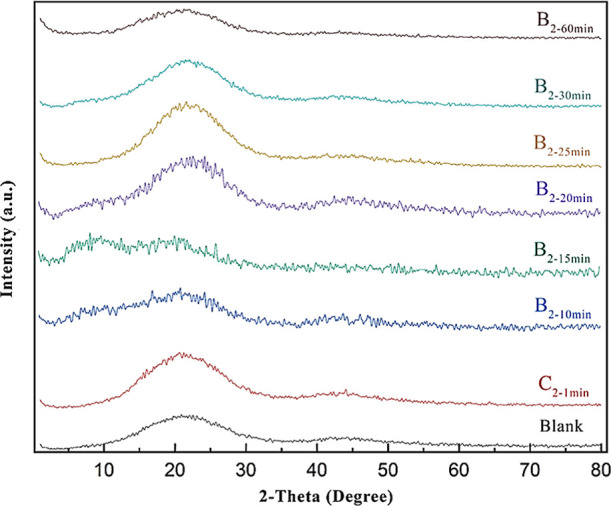
XRD analysis of rPET (blank), C_2–1min_, B_2–10min_, B_2–15min_, B_2–20min_, B_2–25min_, B_2–30min_, and B_2–60min_ prepared by the reactive extrusion and reaction with the CE.

### 3.6 Acid Number Test

We observed that the effect of increasing residence time can influence the IV by two opposing parameters: the CE reaction and degradation. We conducted a study to further confirm this relationship by investigating the impact of increasing the number of acid groups. To evaluate these effects, in this section, a new type of PET was used: PET from water bottles was directly utilized without any cleaning. The water bottles were cut into flakes manually, and the effect of the CE was assessed by measuring viscosity and acid numbers before and after basic hot wash. The hot wash process was carried out on 100 g of PET with 2 L of 5.0% w/w NaOH at 90 °C for 20 min,^41^ followed by a final wash with 100–200 mL of 0.5% w/w HCl solution for neutralization at room temperature. As Table 4 shows and as expected, before the basic hot wash, the acid number was lower and the viscosity of the control sample was higher than that observed after washing. However, the important result of this experiment is shown on the samples that processed with the ESBO CE. As shown in Table 4, the rPET sample that extruded with ESBO showed very close IV for both samples with and without basic hot wash. The IV increase for the PET sample after basic hot wash is much higher than the IV increase for the sample without any treatment, which clearly shows the positive effect of acid groups in terms of ESBO reactivity and effectiveness. During the hot wash process and resulted structural degradation of PET, it was observed that the acid number increases. Consequently, this improves the accessibility and reactivity with the epoxy groups in ESBO, which in turn contributes to the increase in IV. The effect of hot wash on the number of acidic groups appears to be similar to the impact of thermal degradation during extrusion, which also increases the acid number. To further prove this, we have measured the acid number for the rPET (sample 1) before and after continuous extrusion (1 min) that showed values of 16 and 25, respectively. This confirms the increase in acidic groups after extrusion, which is in agreement with this observed trend. As shown in Figure 5, initially, with an increased residence time, a slight decrease in effectiveness was observed. Subsequently, at 10 min, the optimal effect was achieved. After this point, due to the competition between these two parameters—the thermal degradation and the reaction with the CE—the overall impact of the CE decreased, and further degradation occurred. The acid number test successfully confirmed the impact of the end groups on the viscosity change^29^ and showed that the efficacy of the CE increases with an increasing number of acid groups.

**Table 4:** Acid Number of PET from Water Bottles before and after Hot Wash and the IV of them with and without ESBO

treatment	acid number	PET	IV (dL/g)
none (original)	7.3	blank	0.501
		with ESBO	0.548
basic hot wash	9.61	blank	0.470
		with ESBO	0.537

### 3.7 Thermal Analysis

The TGA and DTG results that are shown in Figure 7a,b were conducted in the temperature range of 50 to 650 °C to examine the thermal stability of ESBO and its impact as a CE on the thermal stability of PET samples. The thermal decomposition of ESBO begins at approximately 300 °C, achieving its maximum degradation rate (*T*_max_) at 430 to 440 °C (DTG peak, Figure 7b). This peak degradation temperature is lower than that of the PET polymer, indicating the preliminary decomposition of ESBO, involving glyceride bond scission and epoxy ring opening. However, ESBO exhibits good thermal stability below 300 °C, which is well above the processing temperature of the PET. PET samples undergo a two-step degradation process.^42^ The blank rPET (sample 1) begins to lose weight at approximately 300 °C, with the primary decomposition taking place between 350 and 500 °C,^43,44^ as can be seen in Figure 7a,b. The thermal degradation of the polymer backbone is responsible for this weight loss. The original rPET sample is not as thermally stable as the modified PET samples, PET (C_2–1min_) and PET (B_2–10min_), which contain ESBO. PET (B_2–10min_) exhibits the highest thermal resistance among these samples, as evidenced by a shift in the onset of degradation to higher temperatures and less weight loss in the range of 450–525 °C (Figure 7a). This enhancement is most likely the result of the chain-extending effect of ESBO, which reduces reactive groups at the ends of the chains and increases the molecular weight, thereby enhancing its resistance to thermal degradation. Reducing the active functional groups contributes to decreasing the potential of thermal degradation by these groups, hence ESBO synergistically increases the thermal stability of PET. The decrease in thermal resistance for sample B_2–15min_ compared to sample B_2–10min_ and likely its initial drop in weight (∼1.5% at 300 °C) is aligned with its decreased IV value (Table 3), which in turn is responsible for increasing the number of polar end groups (–OH and–COOH), that are less thermally stable sites and trigger earlier degradation. At 600 °C, the residual weight is relatively consistent across all samples, with a slightly reduced residue observed for B_2–15min_. The DTG curves (Figure 7b) further confirm the enhanced thermal stability of ESBO-modified rPET. While the blank rPET shows its *T*_max_ within the main PET degradation region, B_2–10min_ exhibits a clear shift of the DTG peak to higher temperatures (from 423 to 430 °C) and a lower peak intensity, indicating a reduced degradation rate due to ESBO-induced chain extension. In contrast, B_2–15min_ shows a slightly lower *T*_max_ of 426 °C and broader DTG peak, consistent with its reduced IV. In general, the TGA results suggest that ESBO functions as a thermal stabilizer for PET and that the processing conditions, particularly the extruder residence time, have a significant influence in enhancing the stabilizing effect of the CE.

**Figure 7:**
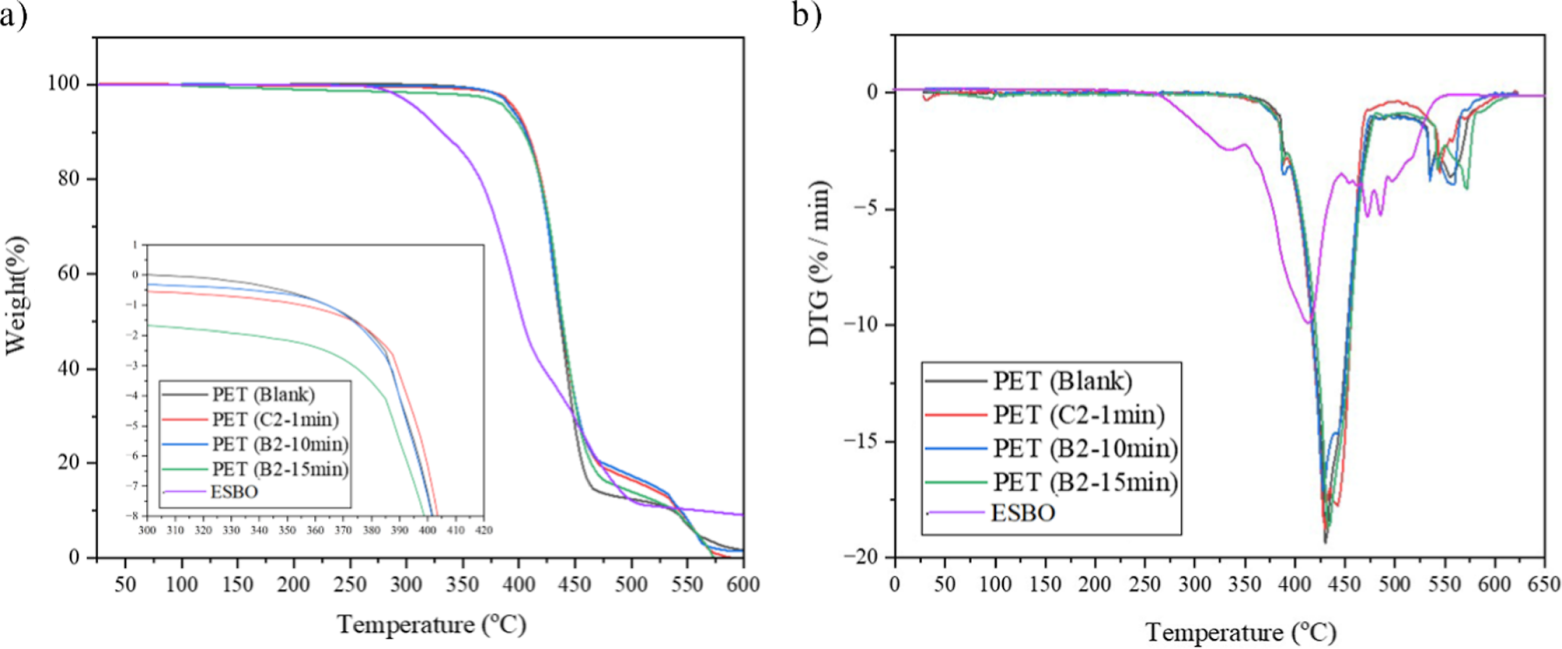
TGA (a) and DTG (b) thermograms of rPET (blank), ESBO, and samples C_2–1min_, B_2–10min_, and B_2–15min_.

The DSC thermograms of ESBO and two rPET samples (C_2–1min_ and B_2–10min_) were recorded in the temperature range of 25–300 °C (Figure 8). ESBO shows a nearly featureless baseline throughout the entire temperature range, indicating the absence of any major phase transition such as degradation and confirming its high thermal and chemical stability up to 300 °C. The DSC analysis of samples C_2–1min_ and B_2–10min_ revealed distinct thermal behaviors. In the temperature range 130–140 °C, corresponding to the exothermic cold crystallization, sample B_2–10min_ exhibited a smaller and broader peak compared to the sample C_2–1min_. This indicates less possibility of cold crystallization for B_2–10min_ compared to the C_2–1min_ in the intermediate temperature range. At higher temperatures, around 250 °C, both samples showed endothermic peaks related to the melting transition, where C_2–1min_ presented a slightly lower melting temperature compared to the B_2–10min_ (251 °C vs 252 °C).

**Figure 8:**
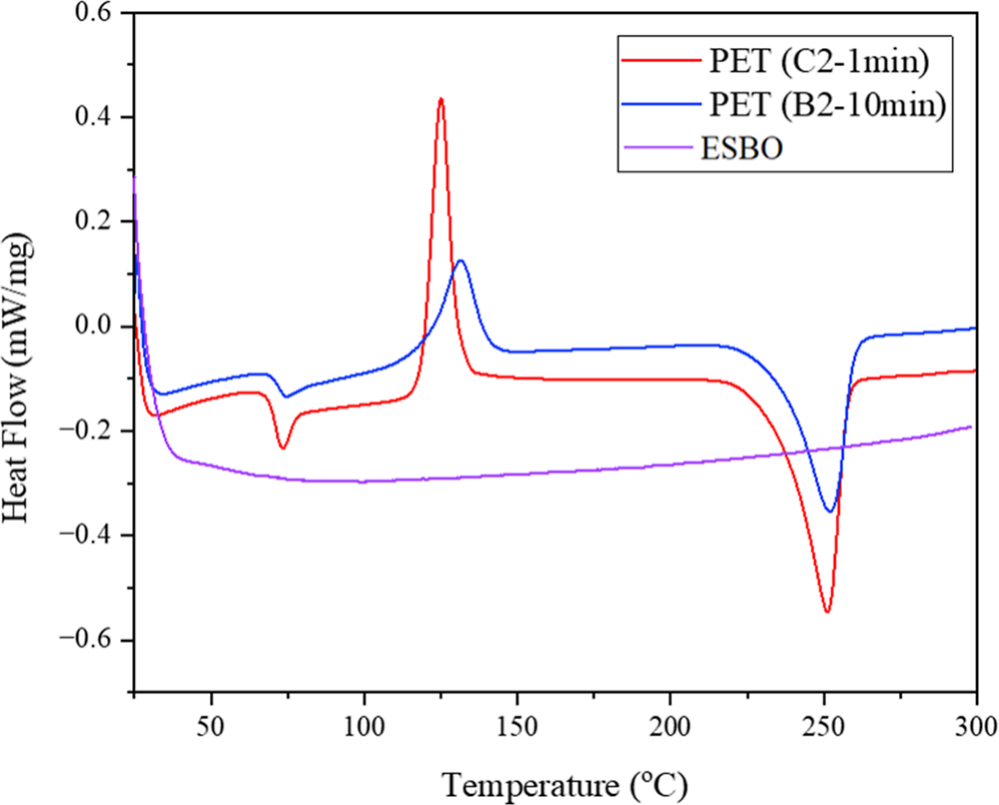
DSC thermograms of ESBO and rPET samples C_2–1min_ and B_2–10min_.

Based on eq 2, the crystallinity of the C_2–1min_ and B_2–10min_ samples was calculated, and the corresponding χ_c_ and *T*_m_ values are presented in Table 5. As can be seen and in full agreement with the XRD results (Figure 6), the B_2–10min_ sample exhibits a noticeable reduction in crystallinity. This reduction is consistent with the expected role of the CE, whose incorporation can hinder chain mobility and interrupt the regular packing of polymer segments, thereby suppressing crystal growth. Furthermore, the decrease in crystallinity may also indicate that the ESBO induced branching and, therefore, have increased the amorphous content of the system.

**Table 5:** Degree of Crystallinity (χ_c_) and Melting Temperature (*T*_m_) for C_2–1min_ and B_2–10min_

sample	χ_c_ (%)	*T*_m_ (°C)
C_2–1min_	13.1	251.1
B_2–10min_	10.8	252.7

### 3.8 Morphological Analysis

To study the effect of ESBO on the morphology of PET, FE-SEM was used. Based on the FE-SEM analysis, the PET samples reacted with the CE exhibit evidence of localized accumulation and morphological heterogeneity, as shown in Figure 9. This suggests that the modification process did not achieve a uniform distribution of the CE throughout the polymer matrix. The observed nonhomogeneity can be primarily attributed to the use of a single-screw extruder, which inherently offers limited shear forces and mixing efficiency, thereby impairing the effective dispersion of the CE. Consequently, the formation of regions with higher molecular weights or cross-linked structures manifests as clusters or masses. Such morphological irregularities indicate that inadequate dispersion and insufficient mixing and shear during processing have led to nonuniform reaction extents, ultimately affecting the microstructure and potentially the mechanical properties of the PET. Therefore, study of the effect of ESBO using a twin extruder with higher mixing efficiency should be perform as our next phase of this project.

**Figure 9:**
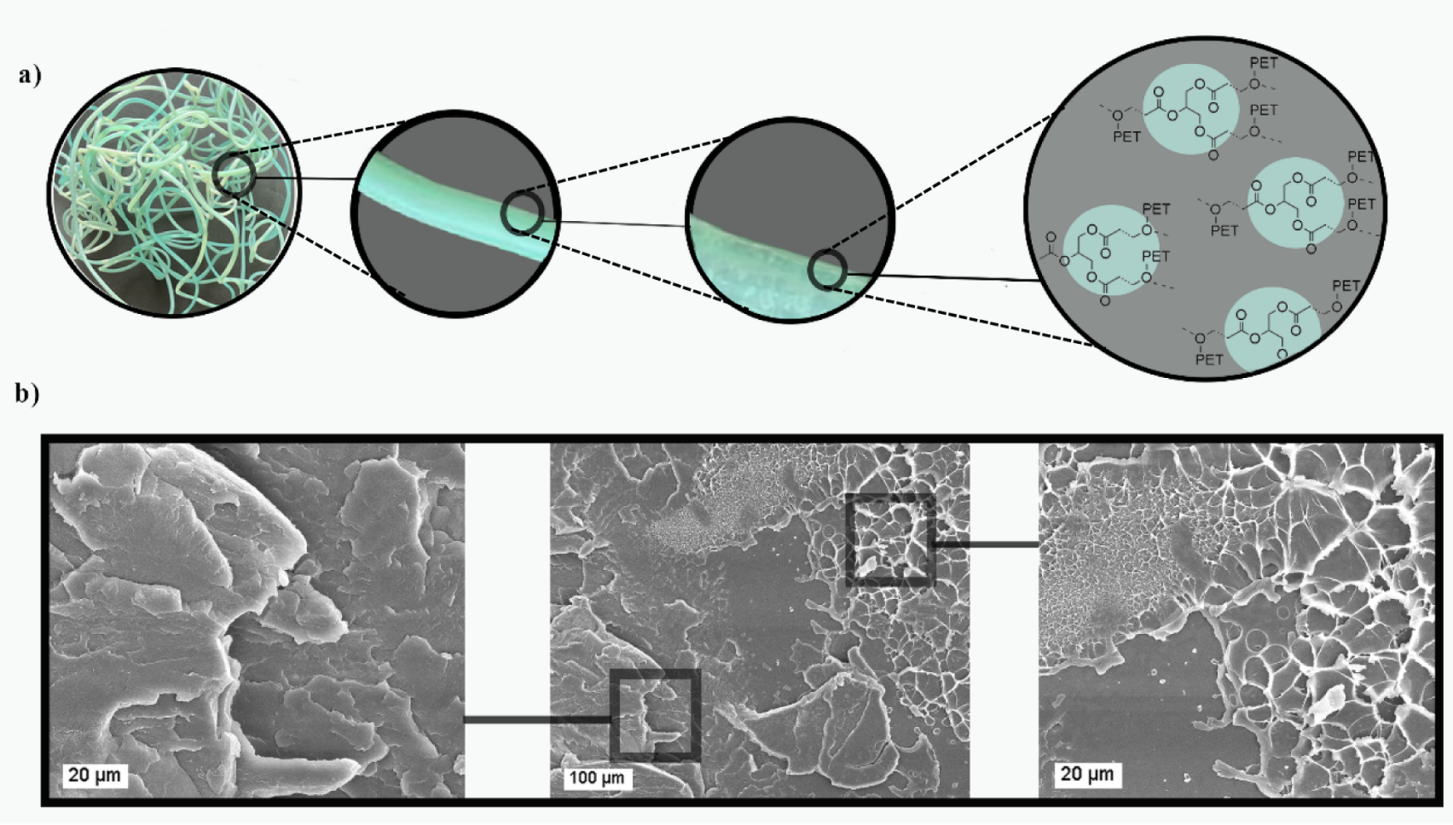
(a) Structural representation illustrating the effect of 2.0 wt % CE on the visible morphology of the extrudate (B_2–10min_). The schematic demonstrates the interaction between PET chains and the CE leading to microstructural modification. (b) Morphological analysis via FE-SEM indicating agglomeration and heterogeneity in the reaction between the CE and PET, attributed to the limitations of the single-screw extrusion process.

### 3.9 Mechanical Analysis

The tensile strength test was performed to compare the mechanical properties of blank rPET to the samples with ESBO, and the results are shown in Figure 10. As obvious in this Figure 10, the B_2–10min_ sample exhibits the highest strength of 57 MPa compared to 51 and 31 MPa for sample B_2–15min_ and blank rPET, respectively. This is in good agreement with the results obtained from the other analyses for these samples. In the B_2–15min_ sample, a reduction in strength is also observed, which aligns with our expectations and its IV, as a longer residence time results in more chain scission and degradation. Overall, the best performance, consistent with the other results, is seen in the B_2–10min_ sample, indicating an acceptable performance of the ESBO potential to enhance the mechanical properties of PET.

**Figure 10:**
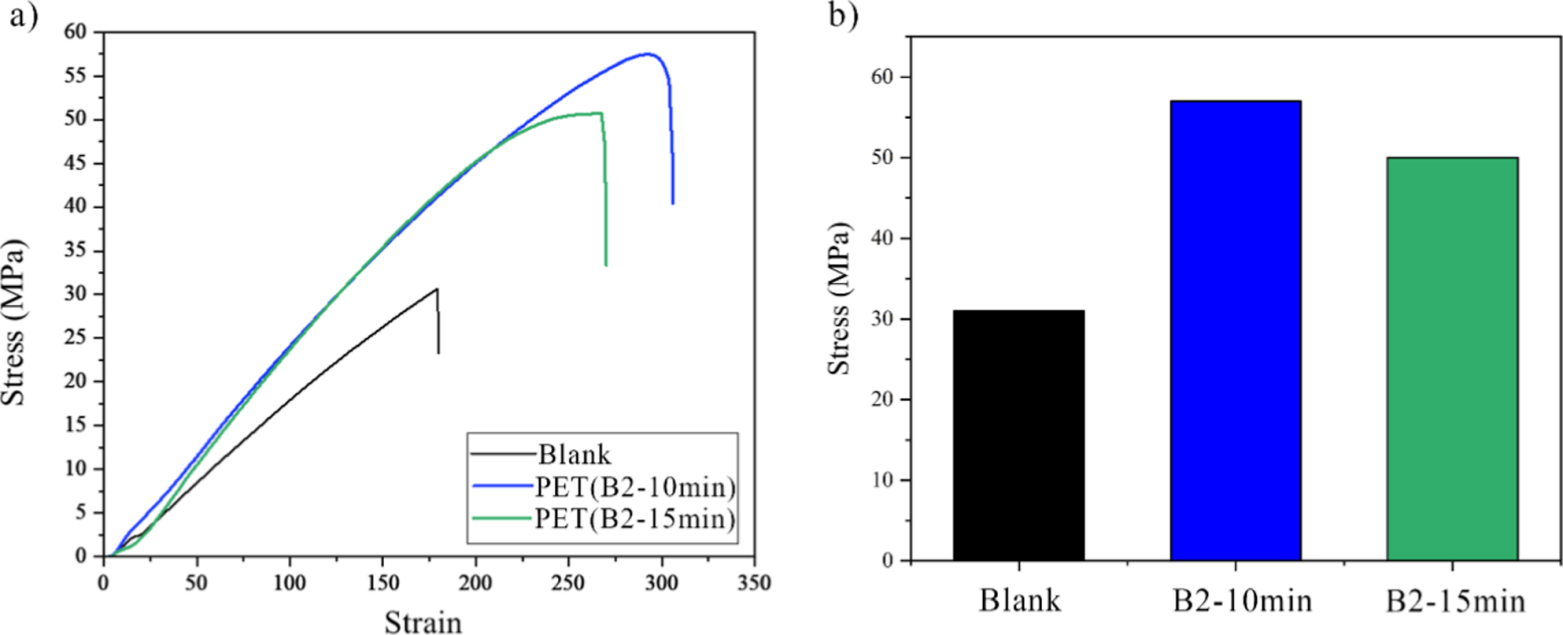
Tensile properties of blank PET and B_2–10min_ and B_2–15min_ samples, (a) stress–strain curve and (b) ultimate tensile strength.

## 4 Conclusion

This study examined the efficacy of a plant-derived CE, ESBO, in a reactive extruder on rPET. The epoxy within the ESBO structure can react with rPET that has degraded in the extruder, thereby compensating for chain scissions caused by high-temperature mechanical recycling. Investigations were conducted on 1 to 4 wt % of ESBO at various residence times in the extruder, with optimal performance observed at 2.0 wt % of ESBO with a residence time of 10 min. Viscometry was employed to assess the efficiency of the ESBO and the reaction conditions on the IV of the rPET, which showed an increase of 0.140 dL/g to approximately 0.680 dL/g for the optimum sample (B_2–10min_). Prolonging the extrusion time more than 10 min, to possibly promote ESBO’s reactivity to access the PET chains end groups, led to thermal degradation that reduced its viscosity. Enhanced mixing at shorter residence times can mitigate the issue and yield better results, while minimizing damage to the PET. The extruder residence time showed two distinct effects. Structural degradation resulted in increased functional end groups on PET, and an enhanced reaction with ESBO, both occurring at different durations and temperatures, requiring careful balancing to achieve the best outcome. Analysis of the acid number and the presence of acidic groups also indicated that an increase in acid groups facilitates reactions between PET and ESBO. The use of ESBO, which is a plant-based CE, demonstrated satisfactory performance at optimized concentrations and can be considered an environmentally friendly method for mechanical recycling of PET. However, the lack of uniform and efficient mixing, which was caused by the limitation of the single-screw extruder, was also evident in the SEM images. It is suggested that this condition can be improved by changing the extruder to a twin-screw extruder or those with higher mixing efficiency. We suggest that a carefully designed extrusion process with a better mixing efficiency can significantly improve the chain extension process and the rPET produce’s properties.
